# The London Hospital's Quinquennial Appeal

**Published:** 1918-02-16

**Authors:** 

**Affiliations:** Chairman. London Hospital, E. 1


### THE LONDON HOSPITAL'S QUINQUENNIAL APPEAL.

### By Viscount Knutsford, Chairman.

### A MILLION HALF-CROWNS.

We publish below Viscount Knutsford's quinquennial appeal for the London Hospital. Lord Knutsford puts it shortly that the "London" has to raise £150,000 a year, with an endowment of only £30,000 a year to depend on.

The present appeal is unique in the sense that it is in part autobiographical, for Lord Knutsford alludes to his tussle with the motor-lorry, which brought in £20,000, by gifts from people who heard about it, and showed their sympathy by sending a cheque to the London Hospital. Next, there is no allusion to, or request for, large cheques as is customary, for Lord Knutsford concentrates his energies upon an effort to collect a million half-crowns by December 1, the birthday of Queen Alexandra, which, if successful, as we hope it may be, will "save the position" and "perpetuate to all time the use of the Finsen Light cure for lupus, first introduced into England" by the President. Many inquiries have reached us as to how the difference between the £30,000 from invested property and the £150,000 which has to be raised each year for the London Hospital is to be made good. At present annual subscriptions produce some £14,000, donations £30,000, boxes £2,000, the King's Hospital Fund £12,000, Hospital Sunday Fund £7,000, Hospital Saturday Fund £900, the private nursing institution, with nurses' and probationers' fees, £5,000, patients' payments £16,000, and legacies £22,000. In the ordinary course it would mean an enormous volume of work to raise one million half-crowns, but with Queen Alexandra as President, and her birthday in front of them, the smallness of the gift, the certainty of receiving a souvenir in the form of a postcard replica half-crown, the generous and united support of the newspapers, Lord Knutsford's proved powers as an advertiser, and his record of quinquennium raisers, the million half-crowns should be lodged at the London Hospital within a month from the issue of the appeal.

The appeal, as worded, should be studied and thought over, for it has caused a number of questions to be raised already, and if every reader of it acts on the impulse of the moment he will write off, propounding his question, and enclosing half-a-crown in the envelope. Then Lord Knutsford will be triumphant indeed, and will be kept fully occupied, we make no doubt, until Queen Alexandra's birthday in December 1918.

### VISCOUNT KNUTSFORD'S APPEAL.

I do not know when I have approached a task with greater misgiving than this one. But it has to be done. We only beg widely every fifth year for the London Hospital, and this year is one such quinquennial.

Since the last quinquennial I have done everything I could think of to keep the hospital going. I have written literally thousands of letters. I have explained to many people the serious difficulties of running a vast unendowed hospital. I have alienated many friends whose friendship towards me was not cheque-deep, and I have been run over by a motor-lorry, which brought in £20,000, but is the sort of thing that cannot be done often.

I do not want to bother your readers with figures. But, shortly put, we have to raise £150,000 a year, with an endowment of only £30,000 a year to depend on.

A large hospital of 1,000 beds is expensive to run, especially nowadays. But it is not only, or even primarily, the "cost per bed" by which a hospital should be judged. The question is whether the money is being properly spent, and its expenditure properly analysed and watched, and as to this I can speak with confidence. Take but one case—the case of nurses: they are underpaid, and too often not too well fed and badly accommodated. We have recently made a further move towards betterment, but this alone has added £4,000 a year to expenditure. Yet it seems to me utterly inexcusable to run a hospital at the expense of its nurses, and we will not do so here.

And so right through. No one who really cares about the work of fighting disease, of helping workers back to work, and mothers and their children to strong life, can hope to do it effectively "on the cheap." If anything is known, or even thought of, which can help to this end, it must be done, and done at the London Hospital. I am not wishing to cry "Wolf!" We mean to keep open doors at the "London" as long as possible, but if we do not get enough money now to carry on during the next five years, some part of the work must go under, or be less well done, and I cannot face this without yet another great effort.

The reputation the London Hospital has made for itself in its 175 years of work needs no words of mine to emphasise it. It has a splendid set of loyal and efficient officers, and a staff of physicians and surgeons second to none in the country. It is equipped to-day to do its work as well as it can be done. It is a miserable thought that all this should be imperilled by the want of money—and no very great sum.

If I can collect a million half-crowns by December 1, the birthday of our President, Queen Alexandra, the position will be saved, and we can, by this collection, perpetuate to all time the use of the Finsen Light cure for lupus, first introduced into England by Her Majesty, who presented the first lamp to the London Hospital. In this department we have treated 100 cases a day for the last fourteen years. So with your kind assistance I send out this appeal for generous help.

Knutsford, Chairman.

London Hospital, E. 1, Feb.

Cheques and postal orders may be sent to me here, crossed, please.

